# Characterization of *Aspergillus aculeatus* β-glucosidase 1 accelerating cellulose hydrolysis with *Trichoderma* cellulase system

**DOI:** 10.1186/s13568-014-0090-3

**Published:** 2015-01-24

**Authors:** Yutaro Baba, Jun-ichi Sumitani, Shuji Tani, Takashi Kawaguchi

**Affiliations:** Graduate School of Life and Environmental Sciences, Osaka Prefecture University, 1-1 Gakuen-cho, Naka-ku, Sakai, Osaka 599-8531 Japan

**Keywords:** *Aspergillus aculeatus*, β-glucosidase, Biomass conversion, *Trichoderma reesei*, Cellulase

## Abstract

*Aspergillus aculeatus* β-glucosidase 1 (AaBGL1), which promotes cellulose hydrolysis by *Trichoderma* cellulase system, was characterized and compared some properties to a commercially supplied orthologue in *A. niger* (AnBGL) to elucidate advantages of recombinant AaBGL1 (rAaBGL1) for synergistic effect on *Trichoderma* enzymes. Steady–state kinetic studies revealed that rAaBGL1 showed high catalytic efficiency towards β-linked glucooligosaccharides. Up to a degree of polymerization (DP) 3, rAaBGL1 prefered to hydrolyze β-1,3 linked glucooligosaccharides, but longer than DP 3, preferred β-1,4 glucooligosaccharides (up to DP 5). This result suggested that there were different formation for subsites in the catalytic cleft of AaBGL1 between β-1,3 and β-1,4 glucooligosaccharides, therefore rAaBGL1 preferred short chain of laminarioligosaccharides and long chain of cellooligosaccharides on hydrolysis. rAaBGL1 was more insensitive to glucose inhibition and more efficient to hydrolyze the one of major transglycosylation product, gentiobiose than AnBGL, resulting that rAaBGL1 completely hydrolyzed 5% cellobiose to glucose faster than AnBGL. These data indicate that AaBGL1 is valuable for the use of cellulosic biomass conversion.

## Introduction

Cellulosic biomass has been most abundant biomass that widely distributed on earth. Cellulose is degraded into monomeric sugar by cellulases, and is appropriate source of biofuels and biochemicals production. However, high crystallinity and insolubility of cellulose makes difficult to degrade to soluble sugar, such as glucose. Cellulose degradation is accomplished by the synergistic action among endoglucanases (E.C. 3.2.1.4), cellobiohydrolases (E.C. 3.2.1.91), and β-glucosidases (BGLs, E.C. 3.2.1.21, Woodward 1991). However it has been known that cellobiohydrolases and BGLs are significantly inhibited by the hydrolysis products such as cellobiose and glucose, and that the product inhibition reduces the overall rate of cellulose hydrolysis (Andric et al. 2010; Xiao et al. 2004). BGLs hydrolyze β-glucosidic bonds to release glucose units from the non-reducing end of β-glucoologosaccharides or glucosides. BGLs are classified in the glycoside hydrolase (GH) family 1, 3, 5, 9, 30, and 116 in the CAZy database (Henrissat 1991; Henrissat and Bairoch 1993,1996; URL: http://www.cazy.org/). In the fungal cellulase system, BGL mainly hydrolyze cellooligosaccharides to glucose on the final step of cellulose degradation. Thus the BGL having high hydrolytic activity is required to forestall the product inhibition by cellobiose against cellobiohydrolases (Du et al. 2010). It has been known that the cellulase mixture secreted by the filamentous fungus *Trichoderma reesei*, which is used for industrial application, has very low activity of BGL, and this problem has been tried to solve by addition of exogenous BGL, for example from *A. niger* (Berlin et al. 2007; Chauve et al. 2010; Dekker 1986; Singhania et al. 2013). Since the BGL is important for cellulase system, the BGL having more powerful activity for hydrolysis and accelerating cellulase system of *T. reesei* is required to promote saccharification of the cellulosic biomass.

*Aspergillus aculeatus* no. F-50 [NBRC 108796] was isolated in soil whose cellulose- and hemicellulose-degrading enzymes effectively hydrolyzed pulp in combination with *T. reesei* (Murao et al. 1979). AaBGL1, a dominant BGL in the culture supernatant of *A. aculeatus* no F-50, was purified and characterized (Sakamoto et al. 1985a,b), and its cDNA was cloned and sequenced (Kawaguchi et al. 1996). AaBGL1 has unique features for hydrolysis of cellooligosaccharides in terms of not only showing high specific activity for cellooligosaccharides with increasing degree of polymerization (DP) up to 5, but also being detected no transglycosylation products on hydrolysis of cellobiose and insoluble cellooligosaccharides by paper chromatography (Sakamoto et al. 1985b; unpublished data). Moreover, Nakazawa et al. reported that *T. reesei* expressing AaBGL1 gene under the control of *xyn3* promoter (X3AB1 strain) exhibited 63- and 25-fold higher BGL activity than that of PC-3-7 strain and X3TB1 strain which expressed *T. reesei* BGL I gene under the control of *xyn3* promoter respectively (Nakazawa et al. 2011). In addition, JN11, which is the crude cellulase preparation from X3AB1, released more glucose than commercially available cellulases, Accellerase 1500 and Cellic CTec from various pretreated biomasses if those have rich hemicelluloses (e.g. NaOH pretreatment; Kawai et al. 2012). Kawai et al. mentioned for these results that JN11 has the best balance of BGL and hemicellulase activities for the degradation of cellulosic biomasses. As described above, AaBGL1 is a useful BGL for biomass conversion. However, AaBGL1 has not been investigated the enzymatic analysis in detail.

There are many fungal BGLs which share high similarity of amino acid sequence with AaBGL1 in the GH family 3. Nevertheless, the reason why AaBGL1 was selected for use in cellulosic biomass conversion, was unclear due to partial investigation of the detailed enzymatic properties. Here we demonstrated the availability of rAaBGL1 by characterization of rAaBGL1, especially substrate specificity and transglycosylation products, and comparing capability to hydrolyze cellobiose with BGL from *A. niger* (AnBGL) which shares high amino acid sequence similarity with AaBGL1.

## Materials and methods

### Strains and medium

*Escherichia coli* DH5αF’ was used as a host for construction of recombinant plasmids. *A. oryzae* niaD300 strain was used as a host for AaBGL1 gene expression.

*A. oryzae* transfomant was cultivated at 30°C with shaking (160 rpm) in Erlenmeyer flasks in the minimal medium (MM) with 5% glucose, 1.5% NaNO_3_, 0.00008% Mo_7_O_24_•2H_2_O, 0.00111% H_3_BO_3_, 0.00016% CoCl•6H_2_O, 0.00016% CuSO_4_•5H_2_O, 0.005% EDTA•2Na, 0.0005% FeSO_4_•7H_2_O, 0.0005% MnCl_2_•4H_2_O, 0.0022% ZnSO_4_•7H_2_O, 0.00013% KCl, 0.00013% MgSO_4_•7H_2_O, 0.00038% KH_2_PO_4_ (pH 6.5) for appropriate days.

### Expression of AaBGL1 gene in *A. oryzae* and purification of recombinant AaBGL1

The AaBGL1 gene was amplified by PCR using *A. aculeatus* genomic DNA as a template, with 5’- aactgcaggcggccgcatcatgaagctcagttggcttg-3’ as a sense primer and 5’-aagcatgctcattgcaccttcgggagc-3’ as an antisense primer. PCR condition is the following thermal settings: 30 cycles of 10 s initial denaturation step at 98°C, followed by 5 s annealing step at 55°C, and 3 min of extension step at 72°C using PrimeSTAR HS DNA polymerase (TaKaRa, Japan). The PCR product was digested by *Not* I and *Sph* I, and inserted into the same sites of *Aspergillus* expression vector, pNAN8142 (Minetoki et al. 2003). The resultant plasmid was named as pNPN-AaBGL1. *A. oryzae* niaD300 strain was transformed with pNAN-AaBGL1 by the protoplast-PEG method (Gomi et al. 1987; Kanamasa et al. 2001). Several transformants was isolated and cultivated to confirm the production of rAaBGL1. The methods of the expression and the purification of rAaBGL1 is previously described (Suzuki et al. 2013). Briefly describing below; *A. oryzae* transformant overexpressing AaBGL1 gene was cultivated in 2.4 L MM liquid medium for 3 days, and the mycelia were harvested and washed with 5 volume 20 mM sodium acetate buffer (pH 5.0). To release rAaBGL1 from cell surface, the mycelia were incubated at 30°C for 2 days in 2.4 L releasing buffer (10 mg/ml cycloheximide, 1 mM benzylsulfonyl fluoride, 0.02% sodium azide in 20 mM sodium acetate buffer (pH 5.0)) with shaking. After releasing AaBGL1 from cell surface, supernatant was obtained by filtration as a crude enzyme. The crude enzyme was applied to a DEAE-TOYOPEARL® 650 M column equilibrated with 20 mM sodium acetate buffer (pH 5.0). rAaBGL1 was eluted with a linear gradient of NaCl (0–0.3 M). The active fractions were collected, added to ammonium sulfate at 30% saturation, and subjected to a Butyl-TOYOPEARL® 650 M column equilibrated with 30% saturation of ammonium sulfate in same buffer. The rAaBGL1 was eluted with a reverse linear gradient of ammonium sulfate (30–0% saturation). After collecting rAaBGL1 containing fractions, the enzyme was precipitated with ammonium sulfate at 80% saturation, dissolved in 20 mM sodium acetate buffer (pH 5.0), and dialyzed in the same buffer. Homogeneity of rAaBGL1 was confirmed by sodium dodecyl sulfate-polyacrylamide gel electrophoresis (SDS-PAGE).

### Purification of BGL from *A. niger*

Glucosidase from *A. niger* (SIGMA-ALDRICH, Co.) was dissolved in 5 ml of 20 mM sodium acetate buffer (pH 5.0). After dialyzing in the same buffer, the same steps of purification as those of rAaBGL1 were performed (Suzuki et al. 2013), followed by gel filtration on HiLaod™ 16/60 Superdex™ 200 pg column (GE healthcare) equilibrated with 20 mM sodium acetate buffer (pH 5.0), and hydrophobic interaction chromatography on Hiprep™ 16/10 Phenyl FF column (low sub; GE healthcare) with reverse linear gradient of ammonium sulfate (30–0% saturation).

### Protein assay

Protein concentration was determined from the absorbance at 280 nm using extinction coefficient (ε) as 162,000 M^−1^•cm^−1^with the exception of the comparison of saccharification between rAaBGL1 and AnBGL*.*

According to the comparison of BGL ability from *A. acuelatus* and *A. niger,* the protein concentration was determined with the Bio-Rad Protein Assay, based on the method of Bradford (Bio-Rad Laboratories). Bovine γ-globulin was used as a standard.

### Enzyme assays

Enzymatic reaction was performed by incubating 100 μl enzyme with 100 μl of 3 mM *p*-nitrophenyl (pNP)-monosaccharides in 100 mM sodium acetate buffer (pH 5.0). Reaction was stopped by adding 2 ml of 1 M Na_2_CO_3_. Released *p*-nitrophenol was quantified by measuring the absorbance at 405 nm using ε as 18.5 mM^−1^•cm^−1^. One unit of BGL activity was defined as the amount of enzyme required for the release of 1 μmol of *p*-nitrophenol per minute from the substrate.

### Effect of temperature and pH on the activity and stability of rAaBGL1

The optimum temperature was determined by incubating rAaBGL1 (6.13 nM) with 1.5 mM *p*-nitrophenyl-β-D-glucopyranoside (pNP-Glc) in 20 mM sodium acetate buffer (pH 5.0) at various temperature (30–70°C) for 10 min. The optimum pH was determined by incubating rAaBGL1 (6.13 nM) with 1.5 mM pNP-Glc in various pH range of buffer (3.3–6.3, sodium citrate buffer; 6.5–7.3, sodium phosphate buffer; 7.0–8.9, Tris–HCl buffer; 8.8–10.7, glycine-NaOH buffer) at 37°C for 10 min. The thermal stability was determined by incubating rAaBGL1 (6.13 nM) at various temperature (30–70°C) for 30 min. After incubation, sample was on ice for 5 min, followed by incubating with 1.5 mM pNP-Glc in 20 mM sodium acetate buffer (pH 5.0) at 37°C for 10 min. The pH stability was determined by incubating rAaBGL1 (61.3 nM) in 100 mM various pH range of buffer (1.8–3.2, glycine-HCl buffer; 3.4–6.0, sodium acetate buffer; 6.5–7.3, sodium phosphate buffer; 7.2–9.1, Tris–HCl buffer; 8.8–10.5, glycine-NaOH buffer) at room temperature for 1 h. After incubation, sample was diluted 10 fold by adding 200 mM sodium acetate buffer (pH 5.0), followed by incubation with 1.5 mM pNP-Glc in 20 mM sodium acetate buffer (pH 5.0) at 37°C for 10 min.

### Inhibition by glucose

For determination of inhibition constant for glucose on AaBGL1, enzymatic reaction was performed by incubation of rAaBGL1 with 0.1, 0.2, 0.3 and 0.4 mM pNP-Glc in the presence of 1.0, 2.5, 5.0, 10.0, 20.0 and 40.0 mM glucose in 20 mM sodium acetate buffer (pH 5.0) at 37°C. Initial rate of released *p*-nitrophenol were measured, and then, *K*_i_ value for glucose on AaBGL1 was calculated with Dixon plot.

For the effect of inhibition by glucose for rAaBGL1 and AnBGL, enzymatic reaction was performed by incubating each enzyme with 1.5 mM pNP-Glc in the presence of 0.05, 0.25, 0.5, 1.0, 2.0, and 4.0% glucose in 100 mM sodium acetate buffer (pH 5.0) at 37°C for 10 min.

### Detection of reaction products by HPAEC-PAD

For detection of enzymatic reaction products, high-performance anion exchange column chromatography (HPAEC) with a pulsed amperometoric detector (PAD) equipped with a CarboPac PA10 guard column (4 × 50 mm) and a CarboPac PA10 analytical column (4 × 250 mm; Dionex Co.) was used. Enzymatic reaction was performed by incubation with equivalent volume of rAaBGL1 (20.0 nM) and each substrate in 20 mM sodium acetate buffer (pH 5.0) at 37°C. Reaction mixture was sampled at appropriate time, and added into equal volume of 0.2 M NaOH. Resultant mixtures were subjected to HPAEC-PAD using mobile phase of 100 mM NaOH with 10 mM sodium acetate. Glucose, cellobiose (Wako Pure Chemical Industries, Ltd.), cellotriose, cellotetraose, cellopentaose, laminaribiose, laminaritriose, laminaritetraose, laminaripentase (Megazyme), gentiobiose, sophorose (SIGMA-ALDRICH, Co.) were used as standards.

### Kinetic analysis

For the kinetic analysis, cellobiose, cellotriose, cellotetraose, cellopentaose, lamianaribiose, laminaritriose, laminaritetraose, laminaripentaose and gentiobiose were used as substrates. Appropriate concentrations of each substrate were mixed with equivalent volume of enzyme in 20 mM sodium acetate buffer (pH 5.0). Every 1 or 2 min, reaction was stopped by adding 50 μl of 1 N HCl, and after 5 min, neutralized with adding 50 μl of neutralizton solution (0.4 N NaOH and 0.8 M Tris). The amount of released glucose was determined by using Glucose CII-Test Wako (Wako Pure Chemical Industries, Ltd.). Kinetic constants were determined using Hanes-Woolf plot according to Michaelis-Menten equation. In the case of disaccharides, *k*_cat_ value was calculated by half of glucose production velocity because one glucodisaccharide molecule composed 2 glucose molecules. Equivalent molar of glucose (G_1_) and G_n−1_ production from G_n_ (n = 3–5) was confirmed at the end point of the reaction by HPAEC-PAD.

For the detection of reaction products by rAaBGL1, laminaribiose, cellobiose, and gentiobiose (25 mM) were reacted with rAaBGL1 (10.0 nM) in 10 mM sodium acetate buffer (pH 5.0) at 37°C. Reaction was stopped by addition of equivalent volume of 0.2 N NaOH and reaction mixtures were analyzed for HPEAC-PAD as described above.

## Results

### Purification and characterization of rAaBGL1

**Figure 1:**
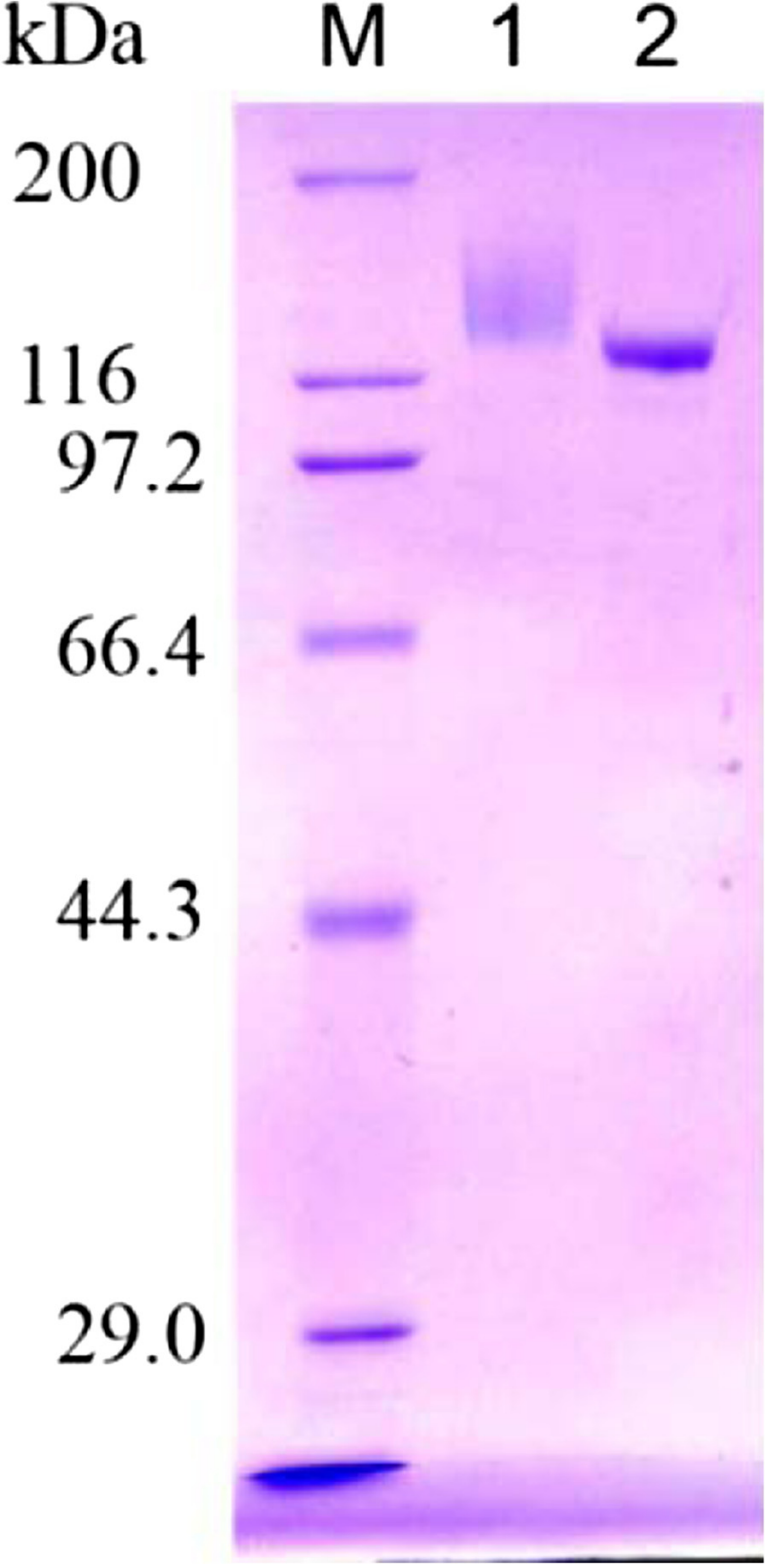
**Purification of rAaBGL1 and AnBGL.** M, molecular weight markaer; 1, AaBGL1; 2, AnBGL.

To investigate the biochemical characterization of rAaBGL1, we purified rAaBGL1 as described in the Materials and methods, and confirmed the homogeneity by SDS-PAGE (Figure 1). The molecular mass calculated from the amino acid sequence was 91.3 kDa, however purified rAaBGL1 was approximately 130 kDa.

**Figure 2:**
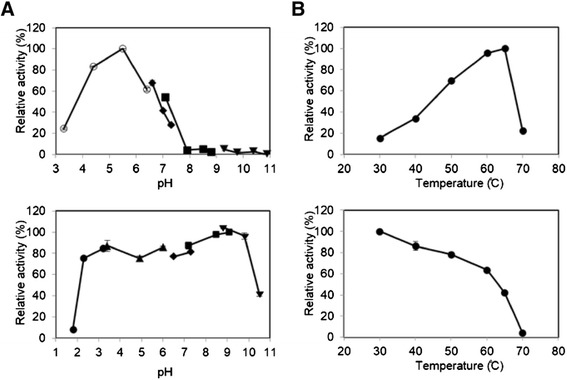
**Effects of pH (A) and temperature (B) on the activity (upper panels) and the stability (lower panels) of purified rAaBGL1.** To determine the effect of pH on the activity and the stability (A), enzyme was incubated with 1.5 mM pNP-Glc for 10 min in 100 mM following buffers: glycine-HCl, pH 1.9–2.8 (closed circle); sodium acetate, pH 3.4–5.9 (closed triangle); sodium citrate, pH 3.3–6.3 (open circle); sodium phosphate, pH 6.4–7.3 (closed diamond); Tris–HCl pH 6.8–8.9 (closed square); Glycine-NaOH pH 9.5–11.0 (closed inveted triangle). To determine the effect of temperature on the activity and the stability (B), enzyme was incubated with 1.5 mM pNP-Glc at 30–70̊C in 100 mM sodium acetate buffer (pH 5.0). Data are expressed at the mean ± the standard deviation of three independent experiments.

Enzymatic properties of rAaBGL1 were determined using pNP-Glc as a substrate. The enzyme was stable between 40–50°C, and in a pH range of 3.0–10.0 with over 80% of its maxmum activity. The optimum temperature was 65°C, and optimum pH was 5.5 (Figure 2).

Specific activity for several pNP-monosaccharides was determined (Table 1). rAaBGL1 was shown highest activity toward pNP-Glc, and slight activity toward pNP-β-D-fucopyranoside, pNP-α-L-arabinofuranoside, pNP-β-D-xylopyranoside, pNP-β-D-galactopyranoside. No activity was detected for pNP-β-D-mannopyranoside, pNP-*N*-acetyl-β-D-glucosaminide.

**Table 1:** **Specific activity of rAaBGL1 for various** ***p*** **-nitrophenyl-β-D-glycopyranosides**

**Substrate**	**Specific activity (U/mg)**
*p*-nitrophenyl-α-L-arabinofuranoside	0.057
*p*-nitrophenyl-β-D-fucopyranoside	0.017
*p*-nitrophenyl-β-D-xylopyranoside	0.428
*p*-nitrophenyl-β-D-glucopyranoside	128
*p*-nitrophenyl-β-D-galactopyranoside	0.006
*p*-nitrophenyl-β-D-mannopyranoside	ND
*p*-nitrophenyl-*N*-acetyl-β-D-glucosaminide	ND

ND:Not Detected.

Substrate specificity and kinetic parameters for natural β-glucooligosaccharides was determined (Table 2). For disaccharide hydrolysis, rAaBGL1 was shown the highest *k*_cat_/*K*_m_ value toward laminaribiose among three disaccharides because of the lowest *K*_m_ value. Cellobiose was not preferable substrate for rAaBGL1 because of the highest *K*_m_ value and lowest *k*_cat_ value. The *k*_cat/_*K*_m_ value for cellooligosaccharides and laminarioligosaccharides were increased up to tetra- and trisaccharide, respectively. AaBGL1 exhibited stationary high *k*_cat_ value for cellopentaose, whereas displayed the lower affinity and turnover number for laminaritetraose and laminaripentaose than laminaritriose.

**Table 2:** **Kinetic parameters of rAaBGL1 for various natural substrates**

**Substrate**	***K***_**m**_ **(mM)**	***k***_**cat**_ **(s** ^**−1**^ **)**	***k***_**cat**_ **/** ***K***_**m**_ **(s** ^**−1**^ **•mM** ^**−1**^ **)**
gentiobiose	0.52 ± 0.02	457 ± 4	873 ± 29
laminaribiose	0.41 ± 0.02	444 ± 17	1080 ± 20
laminaritriose	0.22 ± 0.01	337 ± 3	1550 ± 80
laminaritetraose	0.72 ± 0.03	304 ± 4	423 ± 11
laminaripentaose	1.13 ± 0.01	285 ± 5	251 ± 2
cellobiose	2.06 ± 0.07	354 ± 10	172 ± 3
cellotriose	0.45 ± 0.02	477 ± 3	1060 ± 50
cellotetraose	0.32 ± 0.01	433 ± 4	1340 ± 40
cellopentaose	0.41 ± 0.02	433 ± 9	1070 ± 30

Each value is the mean of triplicate experiments.

### Detection of transglycosylation product

**Figure 3:**
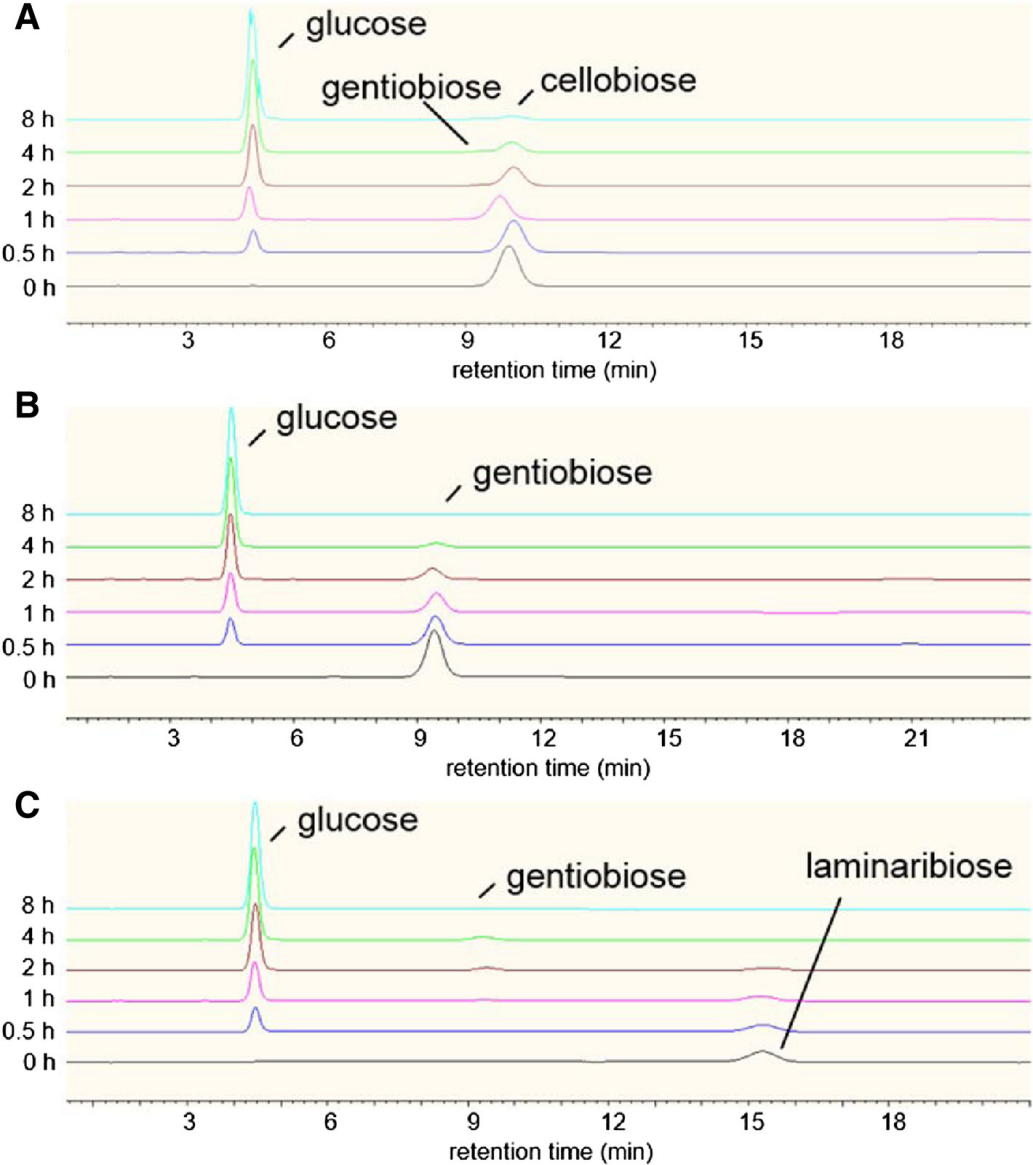
**Hydrolysis of disaccharides by rAaBGL1.** The hydrolysis of 25 mM cellobiose **(A)**, gentiobiose **(B)**, and laminaribiose **(C)** was performed by incubation with 10.0 nM AaBGL1 at 37°C for 8 h. The hydrolysis products of indicating times were analyzed by HPAEC-PAD, and identified by comparison of retention time of each peak with those of standards.

To identify the transglycosylation products by rAaBGL1, the time course of the reaction products using cellobiose, gentiobiose, and laminaribiose as a substrate were analyzed by HPAEC-PAD (Figure 3). In the early stage of the reaction (0–1 h), the reaction product of rAaBGL1 with each substrate was glucose. In the middle stage of the reaction (2–4 h), the reaction products of rAaBGL1 with cellobiose and laminaribiose were glucose and gentiobiose from transglycosylation (Figure 3A,C). In the reaction with gentiobiose, it is expected that gentiobiose was produced as a transglycosylation product as in the case with cellobiose and laminaribiose, because any oligosaccharides other than gentiobiose were not detected (Figure 3B). Thus, these results indicated that gentiobiose was only or main product of transglycosylation by rAaBGL1 under the condition used in this study. In the final stage, the reaction product of rAaBGL1 with each substrate was glucose because of the high hydrolytic activity toward a transglycosylation product, gentiobiose (Table 2).

### Comparison of the saccharification ability between rAaBGL1 and AnBGL on the hydrolysis of 5% cellobiose

**Figure 4:**
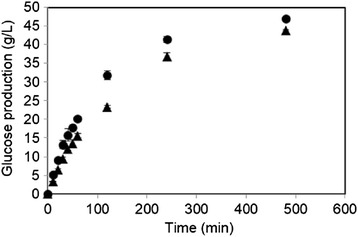
**Comparison of the ability of 5% cellobiose hydrolysis between rAaBGL1 (closed circle) and AnBGL (closed triangle).** Each enzyme was incubated with 5% cellobiose. Released glucose was measured by glucose oxidase method. Data are expressed at the mean ± the standard deviation of three independent experiments.

To evaluate the performance of rAaBGL1, AnBGL which shared 82.4% identity with AaBGL1 (Dan et al. 2000; Seidle et al. 2004) was selected. AnBGL from Sigma-aldrich was purified to homogeneity (Figure 1), and compared the hydrolysis of 5% cellobiose with rAaBGL1 (Figure 4). rAaBGL1 produced glucose faster than AnBGL through the entire reaction time measured. rAaBGL hydrolyzed almost completely 5% cellobiose after 8 h reaction (94.1 ± 0.8%), but AnBGL was not sufficient (82.8 ± 0.4%).

### Glucose inhibition

**Figure 5:**
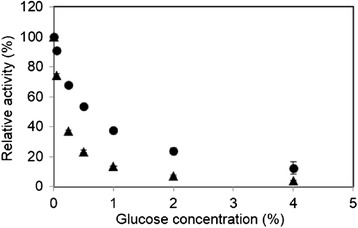
**Comparison of the sensitivity of inhibition by glucose between rAaBGL1 (closed circle) and AnBGL (closed triangle).** Each enzymes were incubated with 1.5 mM pNP-Glc in the presence of 0, 0.05, 0.25, 0.5, 1.0, 2.0, and 4.0% glucose. Released *p*-nitrophenol was measured from the absorbance at 405 nm. Data are expressed at the mean ± the standard deviation of three independent experiments.

Generally, BGL is inhibited by the reaction product, glucose. Therefore we compared the sensitivity to various concentration of glucose on the hydrolysis of pNP-Glc between rAaBGL1 and AnBGL (Figure 5). As a result, rAaBGL1 was lower sensitivity to glucose at all the concentration tested than AnBGL. The *K*_i_ value for gluose on rAaBGL1 used pNP-Glc as a substrate was 9.99 ± 0.94 mM (data not shown).

## Discussion

In this study, we performed detailed investigation of enzymatic properties of rAaBGL1 that have already demonstrated synergistic effects by adding to the cellulase system of *T. reesei*.

Mature AaBGL1 was consisted 841 amino acids, and confirmed secretion in culture supernatant due to possessing signal peptide. The molecular mass calculated from the amino acid sequence was 91.3 kDa, however, purified rAaBGL1 showed approximately 130 kDa by SDS-PAGE analysis (Figure 1). This molecular mass was similar to native AaBGL1 in culture supernatant from *A. aculeatus* (Sakamoto et al. 1985a). Recently, crystalline structure of rAaBGL1, which treated with the endoglycosidase H at undenaturing condition was solved at a 1.80 Å resolution (Suzuki et al. 2013). AaBGL1 has 9 *N*-glycans out of 16 potential *N*-glycosylation sites in the monomer, and *O*-glycosylation was not observed. BGLs from *A. kawachii*, *A. niger* and *A. oryzae* that shared high similarity of amino acid sequence with AaBGL1 have more than 10 potential *N*-glycosylation sites in their amino acid sequence and occur several *N*-glycosylations, in consequence, these BGLs indicated the similar molecular mass with AaBGL1 (Iwashita et al. 1998, 1999; Langston et al. 2006; Seidle et al. 2004).

In previous study, enzymatic properties of authentic AaBGL1 were investigated (Sakamoto et al. 1985b). Thus, we compared enzymatic properties of AaBGL1 between recombinant protein from *A. oryzae* and authentic one. Thermal and pH profiles were almost similar between authentic and recombinant AaBGL1 with exception that rAaBGL1 had higher optimum temperature and thermal stability, and wider range of pH stability than authentic AaBGL1, although assay conditions were different. BGL2, which is probably isoform of AaBGL1 generated by different glycosylation, had higher stability than BGL1. The slight differences of thermal and pH profiles between authentic and recombinant AaBGL1 might be developed from the difference of modification by glycosylation between *A. aculeatus* and *A. oryzae*.

**Figure 6:**
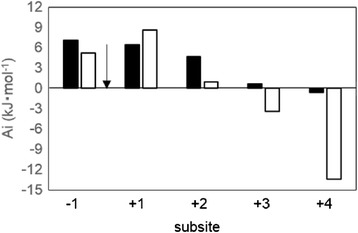
**Subsite affinity map of rAaBGL1 for cellooligosaccharides (black bar) and laminarioligosaccharides (white bar), calculated using the** ***K***_**m**_ **and the** ***k***_**cat**_ **values.** Arrow indicated the cleavage site.

It is known that GH family 3 is composed of β-glucosidase (EC 3.2.1.21), glucan 1,3-β-glucosidase (EC 3.2.1.58), glucan 1,4-β-glucosidase (EC 3.2.1.74), exo-1,3-1,4-glucanase (EC 3.2.1.-), xylan 1,4-β-xylosidase (EC 3.2.1.37), β-*N*-acetylhexosaminidase (EC 3.2.1.52), α-L-arabinofuranosidase (EC 3.2.1.55). In addition, BGLs are divided into three groups on the basis of their substrate specificity, such as aryl-β-glucosidase, aryl- and alkyl-β-glucosidase, and enzymes with broad substrate specificity (Takahashi et al. 2011). Moreover, Harvey et al. classified GH family 3 BGLs into 6 distinct phylogenetic branches by their amino acid sequence similarities (Harvey et al. 2000), and Suzuki et al. mentioned that diverse structures of the subsite +1 makes different substrate specificity in GH family 3 BGLs (Suzuki et al. 2013). However, substrate specificity of AaBGL1 has been investigated only for β-1,4 linked glucan and oligosaccharides (Sakamoto et al. 1985b). Therefore, in this study, the detailed substrate specificity of AaBGL1 was investigated. As a result, rAaBGL1 exhibited high specificity toward pNP-Glc, that is β-glucosidic linkage (Table 1). In kinetic analysis for natural disaccharides, catalytic efficiency increased in the order of cellobiose < gentiobiose < laminaribiose among three β-disaccharides. In all substrates used in this study, rAaBGL1 exhibited highest catalytic efficiency (*k*_cat_/*K*_m_) for laminaritriose due to lowest *K*_m_ value, but longer than laminaritriose, *k*_cat_/*K*_m_ value was decreased because of lowering the affinity and the turnover number. Eukaryotic GH family 3 BGLs have been characterized that there is a tendency for laminaribiose to be the best substrate among the other β-linked disaccharides (Hrmova et al. 2002; Igarashi et al. 2003; Langston et al. 2006; Nakajima et al. 2012; Seidle et al. 2004; Takahashi et al. 2011). On the other hand, for cellooligosaccharides, cellotetraose was the best substrate for rAaBGL1 owing to the stationary low *K*_m_ value and high *k*_cat_ value. In addition, several kinetic analyses of GH 3 BGLs imply that there are three subsites, −1, +1, +2, in the active site, and that subsite +1 is the most important subsite for the substrate binding (Hrmova et al. 2002; Kawai et al. 2004; Nakatani et al. 2010; Yazaki et al. 1997). Subsite map determined by kinetic analysis of rAaBGL1 for cellooligosaccharides hydrolysis, using the method for subsite analysis of exo-acting enzyme (Hiromi et al. 1973), revealed that AaBGL1 had the subsite +2 like other GH family 3 BGLs, but subsite map for laminarioligosaccharides, showed weak affinity at subsite +2 (Figure 6). Previously, it was demonstrated that AaBGL1 had long, aromatic residue-rich cleft in the active site (Suzuki et al. 2013), and these results suggested that this structure contributed to the activity for long chain (DP 4–6) of cellooligosaccharides, but not of laminarioligosaccharides. Thus, rAaBGL1 preferred short chain (DP 2–3) of laminarioligosaccharides and long chain (DP 4–6) of cellooligosaccharides on hydrolysis.

It has not been clear whether rAaBGL1 has transglycosylation activity because of its potent saccharifying activity. During hydrolysis of gentiobiose, we could not detect any oligosaccharide except for substrate (Figure 3B). On the other hand, gentiobiose was detected in the middle stage of hydrolysis for cellobiose and laminaribiose as a substrate (Figure 3A,C). These results indicated that rAaBGL1 has transglycosylation activity, and can produce gentiobiose at least. This result is consistent with the report the glucose molecule at subsite +1 points its C6 hydroxyl to C1 hydroxyl of the glucose molecule at subsite −1 in AaBGL1-glucose complex (Suzuki et al. 2013). In fact, it has been reported that BGL from *A. niger* also produces gentiobiose as one of transglycosylation products (Seidle et al. 2004). Moreover, it has been reported that some BGLs transferred glucose from non-reducing end of substrate to O6 position of non-reducing end of β-linked disaccharides (Kawai et al. 2004; Kono et al. 1999; Seidle et al. 2004; Watanabe et al. 1992). However, we could not clearly identify to produce trisaccharide as a transglycosylation product by rAaBGL1 under the condition used in this study.

In cellulosic biomass hydrolysis using *Trichoderma* cellulase system, cellobiose is accumulated by the dominant enzyme, cellobiohydrolase I that plays an important role for the hydrolysis of crystalline cellulose and by low activity of BGLs. The accumulation of cellobiose causes the lowering of overall activity of cellulase system via the inhibition of cellobiohydrolase activity. Therefore, we attempted to compare the saccharifying activity toward cellobiose between rAaBGL1 and commercially available AnBGL, an orthologue of AaBGL1 (82.4% identity). The optimum pH of AaBGL1 and AnBGL was around 4 (Sakamoto et al. 1985b, Seidle et al. 2004). In this study, we compared the cellobiose hydrolytic activity at pH 5.0, because enzymatic saccharification of cellulosic biomasses was performed at pH between 4.8–5.0 in the many studies (Berlin et al. 2007; Chen et al. 2013; Dashtban and Qin 2012; Harrison et al. 2013; Kawai et al. 2012). As a result, rAaBGL1 hydrolyzed 5% cellobiose faster than AnBGL (Figure 4), because of the difference of the sensitivity for the product inhibition, especially by glucose. In fact, rAaBGL1 was more insensitive to inhibition by glucose than AnBGL at all the glucose concentration tested (Figure 5). *K*_i_ value for glucose of AaBGL1 is 9.99 ± 0.94 mM, on the other hand, that of AnBGL was 3 mM (Seidle et al. 2004). Moreover, the specific activity for a major transglycosylation product, gentiobiose of rAaBGL1 and AnBGL was 170 ± 4 U/mg and 57 ± 1 U/mg, respectively, under the condition of 25 mM gentiobiose in 10 mM sodium acetate buffer (pH 5.0) at 37°C (data not shown). AnBGL exhibited higher *K*_m_ value (1.3 mM) toward gentiobiose than AaBGL1 (0.52 mM), although *K*_m_ value toward cellooligosaccharides of AnBGL was almost similar to AaBGL1 at each optimum pH condition (Seidle et al. 2004). Thus, we concluded that AaBGL1 has high saccharifying activity toward cellobiose compared with AnBGL, because AaBGL1 was insensitive to competitive inhibition by glucose, and has high hydrolytic activity toward gentiobiose, which is transglycosylation product during cellobiose hydrolysis. Recently, the saccahrifying activity of JN11, a crude enzyme preparation from *T. reesei* X3AB1 strain expressing AaBGL1 gene was compared with those of commercially available cellulase, Accellerase 1500 and Cellic CTec using various types of pretreated biomass as substrates (Kawai et al. 2012). Using JN11, especially glucose yield was improved, suggesting that the advantage of JN11 for saccharification of cellulosic biomass resulted in the increase of cellobiase activity by AaBGL1.

In this study, we investigated the biochemical characterization of rAaBGL1 from *Aspergillus acuelatus* no. F-50 strain, and compared the cellobiose-saccharifying activity between rAaBGL1 and AnBGL, an orthologue of AaBGL1. Here we demonstrated the potent cellobiase activity of rAaBGL1 for cellulosic biomass degradation, is combined with the high hydrolyzing efficiency toward gentiobiose derived from transglycosylation and low sensitivity to the product inhibition by glucose.
